# Supplementary material for: The genome sequence of the Arctic Ringlet, Erebia disa (Thunberg, 1791) (Lepidoptera: Nymphalidae)
Source: Wellcome Open Res. 2026 Sep 4;11:587. [Version 1] doi: 10.12688/wellcomeopenres.27700.1 (PMC13755631; doi:10.12688/wellcomeopenres.27700.1)
Supplement: Peer review history

# Reviewer report: Reviewer response for version 1

**DOI:** 10.21956/wellcomeopenres.30491.r171888

**Published:** 2026-11-11

**Authors:** Mario Alejandro Marín

**Affiliations:** 1 Universidad Nacional de Colombia, Medellín, Antioquia, Colombia

This manuscript presents a chromosome-level, haplotype-resolved genome assembly of Erebia disa (Arctic Ringlet) generated within Project Psyche and the Tree of Life Programme. The assembly is of high quality (584 Mb genome, 29 chromosome) and constitutes a valuable resource for future studies of Lepidoptera evolution, conservation, and adaptation to Arctic environments. All sequence data and assemblies are publicly available.

 Overall recommendation

 This is a technically robust Genome Note describing a high-quality chromosome-level assembly that will be a useful resource for the community. The suggested revisions are minor and relate mainly to biological context and interpretation rather than methodological concerns.

 Minor comments

 - Briefly expand the rationale for sequencing E. disa and the anticipated scientific applications of the genome resource.

 - The chromosome-painting analysis is presented descriptively. A short discussion of the evolutionary significance of the observed Merian element patterns would increase the biological value of the manuscript.

 - Provide context for the reported 15,805 protein-coding genes by comparing this value with other Erebia or satyrine genomes.

 - Clarify why individual haplotypes exhibit ~78% k-mer completeness whereas the combined assembly reaches 98.8%, as readers unfamiliar with phased assemblies may find this confusing.

Are the datasets clearly presented in a usable and accessible format, and the assembly and annotation available in an appropriate subject-specific repository?

Yes

Are sufficient details of the sequencing and extraction, software used, and materials provided to allow replication by others?

Yes

Are the rationale for sequencing the genome and the species significance clearly described?

Yes

Are the protocols appropriate and is the work technically sound?

Yes

Reviewer Expertise:

Systematic entomology

I confirm that I have read this submission and believe that I have an appropriate level of expertise to confirm that it is of an acceptable scientific standard.

# Reviewer report: Reviewer response for version 1

**Published:** 2026-09-04

**Authors:** Automated Benchmarking

**Affiliations:** 1 Wellcome Sanger Institute, Hinxton, UK

As this article is a Genome Note, Automated Benchmarking has been used to determine whether the reported assembly passes three defined checks established by the Earth BioGenome Project: the raw and assembled data are publicly available, the Earth BioGenome Project three-part metric is met, and the BUSCO score passes the stated threshold. For Erebia disa (ilEreDisa1), all three checks passed. Data available: pass. ENA study PRJEB80072, raw-read accessions ERR13660088, ERR13670047, and ERR14986712, and assembly accessions GCA_964271725.1 and GCA_964271665.1 are public. Earth BioGenome Project three-part metric: pass. Haplotype 1 is reported as 6.C.Q64, meeting the 6.C.Q40 reference standard. BUSCO: pass. Haplotype 1, which is proposed as the reference assembly for this species, has 98.5% complete BUSCOs (98.0% single-copy and 0.5% duplicated). It meets the target of greater than 90% complete and single-copy BUSCOs. This decision uses values reported in the Genome Note and public accession records.

Reviewer Expertise:

NA

I confirm that I have read this submission and believe that I have an appropriate level of expertise to confirm that it is of an acceptable scientific standard.
